# Study on Performance and Structural Optimization of Concrete Bridge Deck Pavement Materials in Hot and Humid Areas

**DOI:** 10.3390/polym17223072

**Published:** 2025-11-20

**Authors:** Qinghua He, Qun Lu, Qiang Zhang, Chuan Xiong, Chengwei Xing

**Affiliations:** 1 School of Highway, Chang’an University, Xi’an 710064, China; 2 Guangzhou Highway Co., Ltd., Guangzhou 510101, China; 3 Guangzhou Jiaotou Ruijun Real Estate Co., Ltd., Guangzhou 510288, China

**Keywords:** humid and hot regions, concrete bridge deck pavement, mix design, road performance, structural optimization

## Abstract

**Highlights:**

- SMA-13 and AC-13 were identified as optimal pavement materials for hot–humid regions.
- Comprehensive lab tests confirmed the mixtures’ adaptability under extreme climates.
- Epoxy resin significantly improved the interlayer bond strength of the pavement.
- The “SMA-13 + epoxy resin + AC-13”structure enhanced durability and performance.

**Abstract:**

This paper investigated the durability and structural performance of concrete bridge deck pavements under high temperature and high humidity conditions, focusing on three aspects: mix design, road performance evaluation, and structural optimization design. Through Marshall testing, the surface layer material SMA-13 and the middle layer material AC-13 were identified as suitable for hot and humid climates. The former exhibited excellent high-temperature stability and resistance to water damage, while the latter possessed good structural density and load-bearing capacity. A combination of high-temperature, low-temperature, water stability, and impermeability tests was used to systematically evaluate the adaptability of the mixture in hot and humid environments. Furthermore, the performance of different interfacial bonding materials was analyzed through interlaminar pull-out and direct shear tests. The results revealed that the incorporation of epoxy resin notably enhanced the interlayer bond strength and overall durability of the pavement system in hot and humid environments. The proposed “SMA-13 + epoxy resin + AC-13” configuration demonstrates promising potential for improving the mechanical performance and service life of concrete bridge deck pavements.

## 1. Introduction

With the continuous increase in traffic volumes and the growing proportion of heavy vehicles, the mechanical loads borne by bridge structures have risen substantially [1]. As a result, early stage pavement defects are occurring more frequently, which seriously affect bridge durability and driving safety. Serving as the direct interface between vehicles and bridges, concrete bridge deck pavements play multiple essential roles, including waterproofing, crack resistance, and wear resistance. Therefore, the material properties and structural configuration of deck pavements are of great importance to the overall mechanical behavior and service performance of bridge systems. Over the past decades, extensive research at home and abroad has been devoted to the study of concrete bridge deck pavement materials and structures [2,3]. In particular, the wide application of asphalt concrete in bridge pavements has provided valuable design concepts and technical approaches for improving pavement performance.

Internationally, the Japan Road Association has proposed a multilayer pavement design for reinforced concrete bridges, composed of an asphalt layer combined with sheet-type waterproofing materials, asphalt rubber adhesive, and a concrete substrate to form an integrated waterproof and wear-resistant composite structure [4]. In Denmark, a multilayer asphalt concrete system is adopted, consisting of an epoxy resin primer, a modified asphalt membrane, a 15–20 mm open-graded asphalt concrete protective layer, and a 40 mm modified asphalt concrete bonding and wearing layer [5]. Germany employs a similar system but introduces an air-venting layer to relieve interfacial stress while omitting the protective layer [6]. In France, design specifications require the inclusion of a waterproof layer, recommending coatings or membranes made of high-molecular polymers [7]. Typically, such bridge deck pavement systems adopt a double-layer configuration, in which the upper layer bears the traffic load and the lower layer provides waterproofing and isolation to ensure structural stability and long-term durability.

In China, the materials and structural design of cement concrete bridge deck pavements remain relatively simple, often derived from conventional highway pavement structures [1]. Dense-graded asphalt concrete mixtures (such as AC and SMA) are commonly used as surface and protective layers, with thicknesses generally between 30 and 50 mm [8]. Various waterproof adhesives, including modified emulsified asphalt, hot-melt modified asphalt, and reactive resins, are applied in conjunction with these layers. However, a significant stiffness mismatch exists between the pavement layer and the bridge deck. The elastic modulus of the deck usually exceeds 30,000 MPa, while that of asphalt concrete ranges from 800 to 1600 MPa. This disparity causes interfacial stress concentration and often results in insufficient interlayer bonding strength [9]. Moreover, the thin pavement layer and the high-temperature rolling during construction can soften or even damage the waterproofing layer, leading to voids, cracks, and localized bulging in the pavement. These defects severely reduce the durability and service performance of bridge decks.

Hot and humid regions, which represent a typical climatic condition in southern China, impose even greater challenges on bridge pavement materials. The average annual temperature generally ranges from 25 °C to 30 °C, with summer highs exceeding 45 °C. Annual precipitation reaches 1500–2500 mm, and relative humidity remains above 75% [10]. Such environmental conditions place extremely high demands on the high-temperature and moisture resistance of pavement materials. Under these conditions, the softening point of asphalt-based waterproof materials (normally between 55 °C and 65 °C) can be easily reached or exceeded by the actual surface temperature of bridge decks, significantly reducing waterproofing performance. Meanwhile, frequent rainfall promotes water infiltration, resulting in moisture-induced damage and freeze–thaw deterioration. Typical distresses such as asphalt bleeding, surface peeling, cracking, and interlayer debonding become more severe, ultimately shortening service life and compromising driving safety. At present, most research on bridge deck pavements has focused on temperate or arid climates, with limited systematic studies addressing material performance and structural optimization for hot and humid environments. Consequently, bridge pavements in such regions often exhibit service lives of less than 5–7 years, far shorter than their design expectations [11]. Frequent maintenance not only increases the life cycle costs, but also disrupts traffic operations and elevates safety risks.

In light of these challenges, this study focused on the performance characteristics of concrete bridge deck pavement materials in hot and humid regions. Through an integrated approach combining mixture design, pavement performance testing, and structural mechanics evaluation, the study evaluated the high-temperature stability, moisture resistance, and interlayer bonding performance of representative material. The synergistic effects between material properties and structural configurations under hot and humid environmental conditions were systematically analyzed. Based on these findings, an optimized pavement structure was proposed to enhance high-temperature stability, waterproofing capacity, and overall durability. The research aims to provide theoretical guidance and practical solutions for improving the long-term performance of bridge deck pavements in hot and humid climates.

## 2. Materials and Methods

### 2.1. Raw Materials

The performance of asphalt mixtures is closely linked to the intrinsic properties of their constituent materials. In this study, an SBS-modified asphalt binder and a well-graded natural crushed aggregate were employed. The fundamental physical and mechanical properties of these raw materials are detailed in Table 1 and Table 2. The epoxy resin and emulsified asphalt were procured from the local construction material market. The cured epoxy resin exhibited a tensile strength of 47.6 MPa, with a resin-to-hardener ratio of 3:1 as recommended by the supplier, and the diluent content was limited to 5%. Following the literature recommendations, the optimal application rate of the epoxy resin tack coat was 0.4 kg/m^2^. The emulsified asphalt used was a slow-setting cationic emulsion (CSS-1, commercial grade) with a residual asphalt content of 63%. Its optimal application rate was determined as 0.25 kg/m^2^.

### 2.2. Experience Methods

#### 2.2.1. Mix Design

The Marshall design method was used to design the material composition of common asphalt mixtures for the top layers (SMA-10 and SMA-13) and middle layers (AC-13, AC-16, and AC-20) of concrete bridge deck pavements. The aggregate gradation and mix compositions of these mixtures are presented in Table 3. Standard Marshall specimens were compacted 75 times on each side using a Marshall compactor. The specimens had a diameter of 101.6 ± 0.2 mm and a height of 63.5 ± 1.3 mm. The specimens were placed between two semicircular indenters and loaded at the specified temperature and speed.

#### 2.2.2. Road Performance Test

- High-temperature performance test

**Figure 1:**
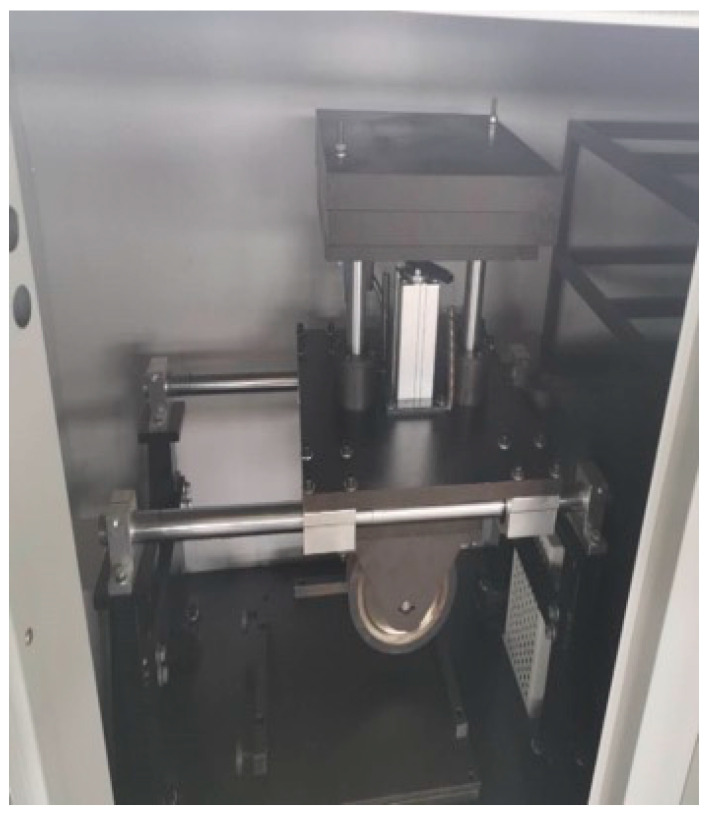
High-temperature performance test.

The high-temperature performance of the asphalt mixture was evaluated using the rutting test in accordance with JTG E20-2011. Asphalt mixtures were prepared following the designed gradation and asphalt content, and specimens were fabricated using a rolling compaction method to achieve the target density and thickness of 50 mm. The rutting test was conducted at a temperature of 60 °C under a 0.7 MPa wheel load and a loading frequency of 42 passes/min using a wheel tracking device (Figure 1). Prior to testing, specimens were conditioned in a temperature-controlled chamber at 60 °C for 2 h to ensure uniform thermal equilibrium [12]. The equipment was started, and the loading wheel was reciprocated across the specimen surface. The change in rutting depth over time was recorded during the test to evaluate the asphalt mixture’s rutting resistance. During the test, the wheel reciprocated over the specimen surface, and the rut depth was continuously recorded. The dynamic stability (DS), representing the mixture’s resistance to permanent deformation, was calculated as the number of load passes per millimeter of rut depth. A higher DS value indicates better high-temperature stability and deformation resistance of the asphalt mixture.

- Low-temperature performance test

**Figure 2:**
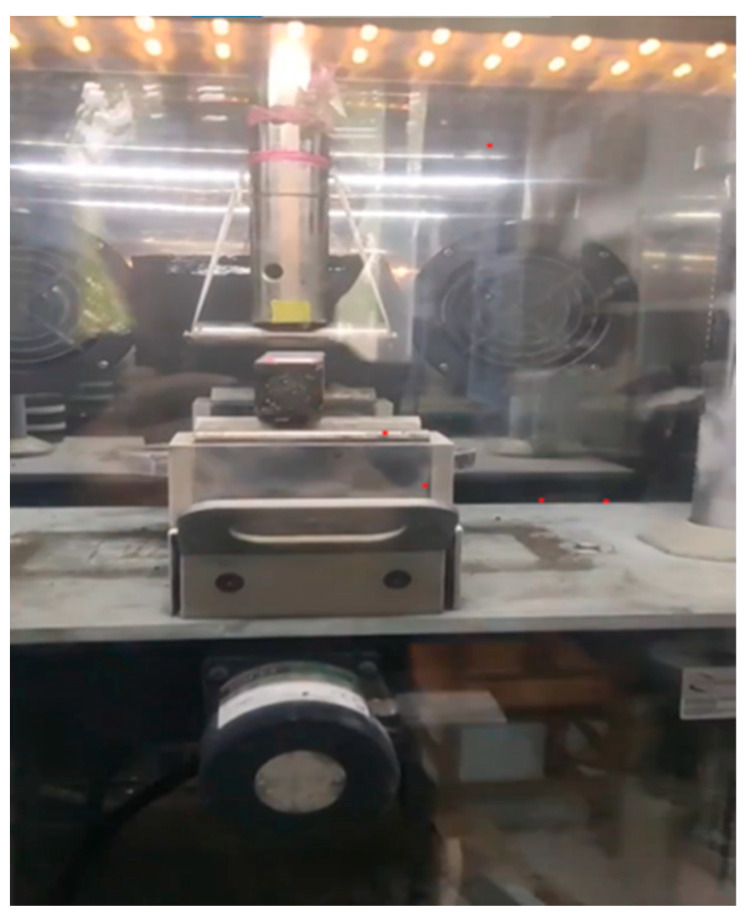
Low-temperature performance test.

The low-temperature crack resistance of the asphalt mixture was evaluated using the three-point bending test in accordance with JTG E20-2011. Small beam specimens with dimensions of 250 mm × 30 mm × 35 mm were fabricated by the rolling compaction method and subsequently conditioned in a low-temperature chamber at –10 °C for 4 h to achieve uniform thermal equilibrium [13]. The test was performed using a Universal Testing Machine (UTM) under a loading rate of 50 mm/min (Figure 2). A vertical load was applied at the midspan until specimen failure occurred. The maximum bending strain was determined using the recorded load–deflection data, which characterizes the material’s resistance to cracking at low temperatures.

- Water stability test

**Figure 3:**
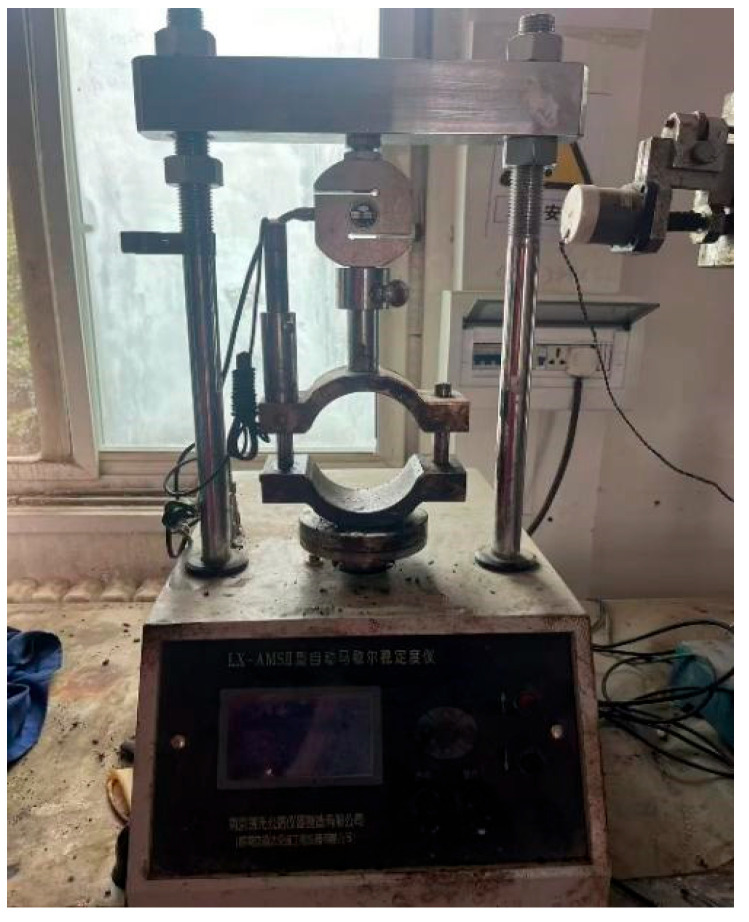
Water stability test.

As shown in Figure 3, in the Marshall immersion test, following the test method specified in JTG E20-2011, the specimens were immersed in a constant-temperature water tank at 60 °C for 48 h. The Marshall stability test was then conducted to measure the stability MS1. This stability MS1 was compared with the stability MS obtained in this section, and the stability ratio (IRS) was calculated and expressed as a percentage. A higher IRS indicates a lower impact of water on the asphalt mixture and a higher water stability [14].

- Water permeability test

**Figure 4:**
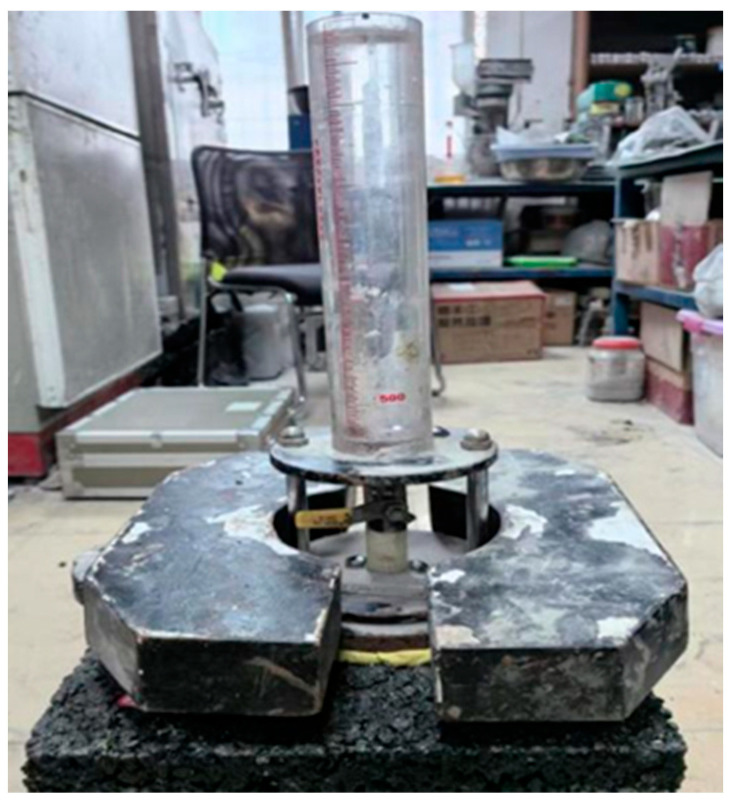
Water permeability test.

According to the JTG E20-2011 test specification, standard rutting plate specimens were first prepared. The specimens were placed on a stable surface, and the test area was sealed with a plastic ring. The specimen was then positioned above a water collection container, and the permeameter was aligned with the test point and pressurized (Figure 4). Water was introduced into the burette while venting to ensure the absence of air bubbles. The time required for the water level to drop from 100 mL to 500 mL, or the volume of water passing within 3 min, was recorded. Three specimens of the same material were tested, and the average value was taken as the measured permeability coefficient.

#### 2.2.3. Structural Optimization Design

- Fabrication of composite rutting plate specimens

To investigate the effects of base layer thickness and mixture type on the rutting resistance of composite asphalt structures, double-layer composite rutting plate specimens were prepared in accordance with the requirements of JTG E20-2011. First, AC-13C or AC-20C base mixtures were hot-mixed and compacted in a standard rutting plate mold. After compaction and cooling, the base layer was transferred to a mold corresponding to the dimensions of the upper layer. An emulsified asphalt waterproof bonding layer was then uniformly applied to the base surface, with application rate and construction conditions controlled according to JTG F40-2004. Immediately after bonding treatment, the SMA-13 surface layer was placed and compacted to form an integrated composite specimen. Three structural configurations were designed: 4 cm SMA-13 + 4.5 cm AC-13C, 4 cm SMA-13 + 6.0 cm AC-13C, and 4 cm SMA-13 + 6.0 cm AC-20C. All specimens were left at room temperature for 24 h prior to the rutting tests.

- Fabrication of pull-out and direct shear specimens

To further analyze the interlayer bonding performance of thin structures, cylindrical specimens were prepared based on the 4.0 cm SMA-13 + 4.5 cm AC-13C configuration using different types of waterproof bonding layers. The bonding layers included emulsified asphalt, hot-modified asphalt, and epoxy resin, with the objective of evaluating their influence on interlayer bond strength. The composite slabs were first fabricated following the same procedure as for the double-layer rutting plate specimens. Core samples with a diameter of 100 mm were then extracted from the slabs to serve as specimens for subsequent pull-out and direct shear tests.

- Pull-out test

**Figure 5:**
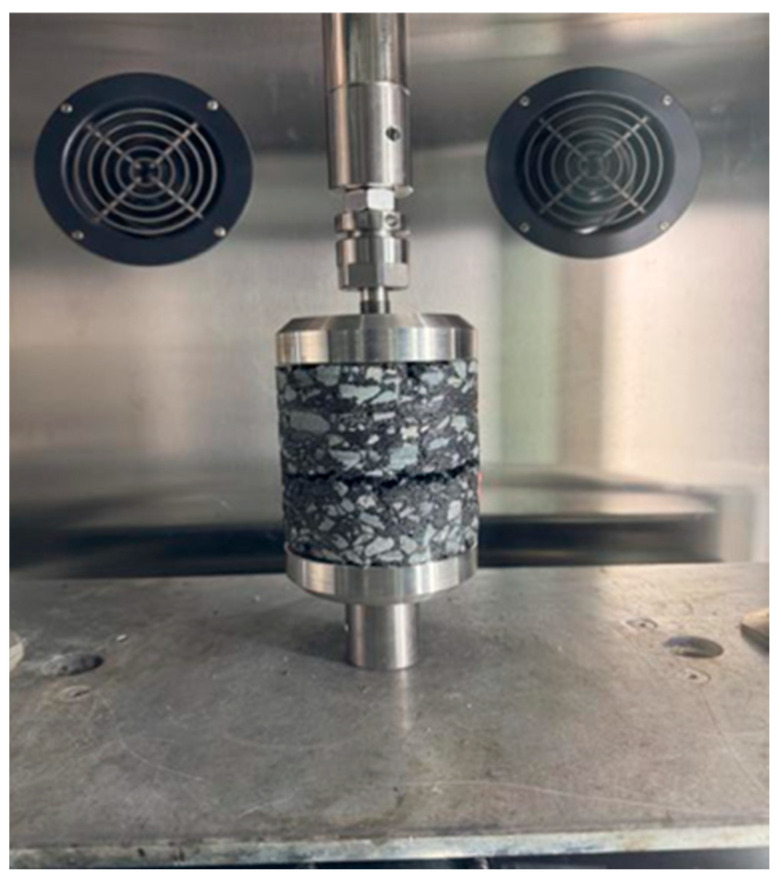
Pull-out test.

The pull-out test was conducted by applying a vertical tensile force perpendicular to the interlayer until complete separation occurred, and the maximum load was recorded to calculate the interlayer pull-out strength. This method provides a direct evaluation of the intrinsic bonding capacity of the adhesive materials. The test was performed using a UTM servo-controlled universal testing machine with a load capacity of 30 kN, as shown in Figure 5.

The procedure was as follows: the pull-out heads were firmly bonded to the top and bottom surfaces of the prepared specimens using epoxy resin. After the adhesive had cured, the specimens with attached pull-out heads were placed in an oven at the specified temperature for 4 h. The specimens were then removed, mounted, and aligned in the testing device. Displacement-controlled loading was applied at a rate of 10 mm/min until complete interlayer failure occurred. The pull-out strength calculation formula is shown in Equation (1).

(1) $$\sigma =\frac{F}{S}$$

where σ is the pull-out strength (MPa), F is the maximum tensile force (N), and S is the interlayer stress area (mm^2^)

- Direct shear test

**Figure 6:**
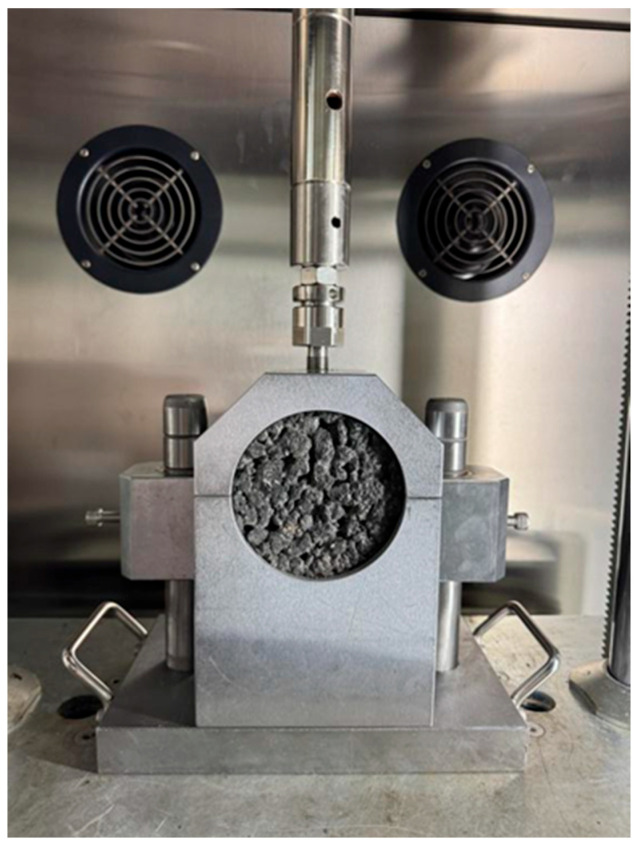
Direct shear test.

The direct shear test was performed by applying a load parallel to the interlayer plane until complete shear failure occurred. The maximum load was recorded and used to calculate the direct shear strength. The test was carried out with a UTM servo-controlled universal testing machine with a load capacity of 30 kN, as shown in Figure 6. The test procedure is as follows: the shear specimens were conditioned in an oven at the specified temperature for 4 h and then fixed in a dedicated direct shear test fixture. Displacement-controlled loading was applied at a rate of 10 mm/min until complete interlayer shear failure occurred. The peak load during the test was recorded and used to calculate the direct shear strength according to Equation (2).

(2) $$\tau =\frac{F}{S}$$

where τ is the direct shear strength (MPa), F is the maximum pressure value (N), and S is the interlayer stress area (mm^2^)

## 3. Results and Discussion

### 3.1. Gradation Design

To evaluate the mechanical performance and structural suitability of asphalt mixtures with different gradation types, Marshall tests were conducted on five representative mixtures (SMA-10, SMA-13, AC-13, AC-16, and AC-20). The results are presented in Table 4. The indices examined provide a comprehensive assessment of mixture compactness, durability, and mechanical behavior.

SMA-10 and SMA-13, commonly applied as surface courses for bridge decks and heavy-load pavements, both demonstrate satisfactory compactness and load-bearing capacity. The air voids of SMA-13 (4.3%) were slightly higher than those of SMA-10 (3.5%), indicating a relatively looser structure; however, the value remained within the recommended specification range (3–5%). This structural porosity enhances resistance to moisture damage and improves surface drainage. In terms of stability, SMA-13 reached 11.39 kN, markedly exceeding SMA-10 (9.22 kN), thereby exhibiting stronger resistance to rutting deformation under heavy traffic loads. The balance between its bulk density (2.415 g/cm^3^) and VMA (16.5%) contributes to a well-formed asphalt–aggregate bonding, ensuring reliable long-term performance.

As a typical intermediate course mixture, AC-13 demonstrated favorable compactness, with an air void content of 4.8%, significantly lower than AC-16 (6.9%) and AC-20 (7.2%). This indicates its superior ability to inhibit water ingress and limit compressive deformation during load transfer, making it suitable as a transitional layer between upper and lower courses. Mechanically, AC-13 achieved a stability of 11.04 kN, comparable to SMA-13 and distinctly higher than AC-16 (8.55 kN) and AC-20 (9.35 kN). This reflects its strong load-bearing capacity and structural stability, supporting its application in medium-traffic pavements or in structurally sensitive areas of the upper base. Notably, despite the relatively high maximum theoretical relative density of AC-20 (2.548), its asphalt saturation is only 46.30%, suggesting insufficient binder distribution. Consequently, its water resistance and flexibility may be compromised, rendering it more suitable for lower pavement layers or temporary structures rather than surface applications.

In summary, SMA-13 demonstrates superior performance in terms of compactness, stability, and asphalt saturation, offering a favorable balance between deformation resistance and durability. These attributes make it particularly appropriate for critical regions subjected to high temperatures, heavy loads, or bridge deck paving. AC-13 achieves an effective compromise between load-bearing capacity and compactness, thereby serving as an optimal intermediate course material that facilitates load transfer, stress dispersion, and structural buffering. The combination of SMA-13 as the surface course and AC-13 as the underlying layer can significantly enhance the integrated performance of pavement systems, strengthening their resistance to fatigue, moisture-induced damage, and long-term deterioration, thus ensuring strong engineering applicability.

### 3.2. Road Performance Test

#### 3.2.1. High-Temperature Performance

**Figure 7:**
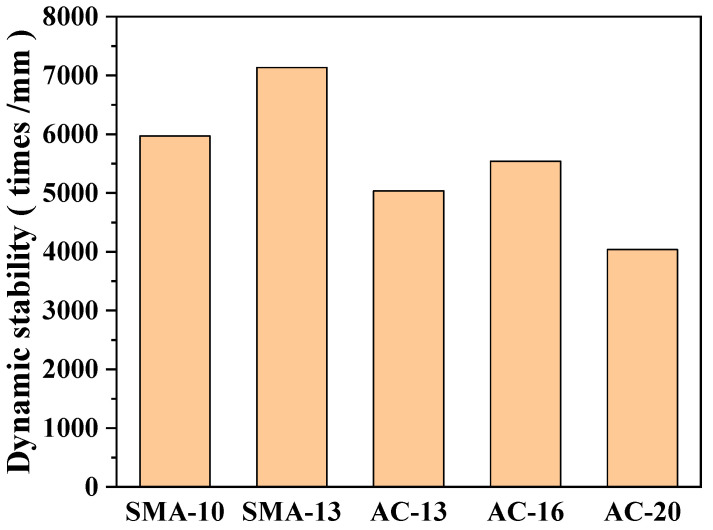
Rutting test results.

To evaluate the rutting resistance of asphalt mixtures with different gradation types under high-temperature conditions, wheel-tracking tests were conducted to determine their dynamic stability. The results are summarized in Figure 7. According to JTG F40-2004, the dynamic stability of asphalt mixtures should not be less than 1800 passes/mm. All five mixtures examined in this study satisfied this requirement. Among the mixtures, SMA-13 exhibited the highest dynamic stability, reaching 7132 passes/mm, which is significantly superior to the other gradations and indicative of exceptional rutting resistance. This outstanding high-temperature stability can be attributed to the stone-on-stone skeleton formed by the coarse aggregate framework in SMA mixtures, which provides a strong interlocking structure [15]. Furthermore, the asphalt–fiber composite binder system enhances the mixture’s capacity to resist shear deformation at elevated temperatures. In comparison, AC-13 achieved a dynamic stability of 5037 passes/mm. Although slightly lower than SMA-13, it still demonstrated a satisfactory level of high-temperature stability, making it suitable for use as an intermediate course. AC-16 and AC-20 exhibited moderate performance, with dynamic stability values of 5542 passes/mm and 4039 passes/mm, respectively. These results suggest that they are better suited for secondary structural layers, such as lower base or sub-base courses, where they primarily serve to support and distribute the loads transmitted from the upper layers.

#### 3.2.2. Low-Temperature Performance

**Figure 8:**
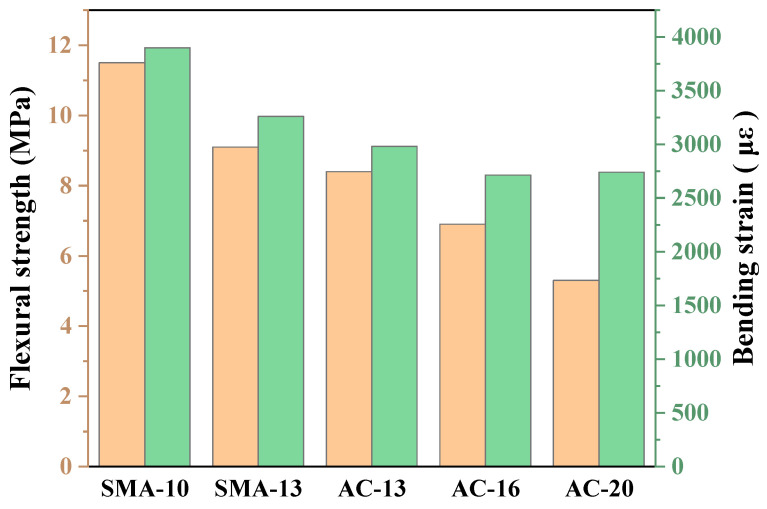
Three-point bending beam test results.

The three-point bending beam test at low temperature is a critical method for assessing the crack resistance of asphalt mixtures. Bending strength reflects the material’s fracture resistance, while bending strain serves as an indicator of its flexibility. According to JTG F40-2004, the bending strain should not be less than 2500 με. As shown in Figure 8, all mixtures evaluated in this study satisfy this requirement. SMA-10 demonstrated the most favorable low-temperature performance, achieving a bending strength of 11.5 MPa and a strain capacity of 3900 με. These results indicate not only excellent crack resistance but also strong resilience against thermal contraction-induced deformation [16]. SMA-13 exhibited a bending strength of 9.1 MPa and a strain of 3261 με, reflecting a desirable balance of flexibility and crack propagation resistance. AC-13 showed comparatively lower low-temperature performance, with a bending strength of 8.4 MPa and strain of 2981 με, though still maintained an adequate level of crack resistance. In contrast, AC-16 and AC-20, characterized by looser aggregate structures and higher air void contents, exhibited increased brittleness under low-temperature conditions. Their bending strengths were limited to 6.9 MPa and 5.3 MPa, respectively, suggesting that these mixtures are more appropriate for application in regions with smaller temperature fluctuations.

#### 3.2.3. Water Stability

Water stability is a key indicator of asphalt mixture resistance to water damage. The immersion Marshall test was used to analyze the mixture’s strength retention in a water environment. According to the specification, the residual stability should be no less than 75%, and all samples met this requirement. Specific data are shown in Table 5. The SMA-13 mixture had an initial stability of 11.39 kN, and after immersion, it maintained a stability of 10.64 kN, with a residual stability of 93.4%, demonstrating exceptional resistance to water damage. This is attributed to its high density and asphalt–fiber synergistic bonding system, which effectively inhibits water erosion [17]. The AC-13 mixture had a residual stability of 88.5%, slightly lower than that of SMA mixtures, but still at a high level, indicating that its structure is still water-stable. The values for AC-16 and AC-20 were 83.0% and 80.7%, respectively, indicating that resistance to water erosion decreases with increasing particle size and porosity.

#### 3.2.4. Water Permeability Coefficient

**Figure 9:**
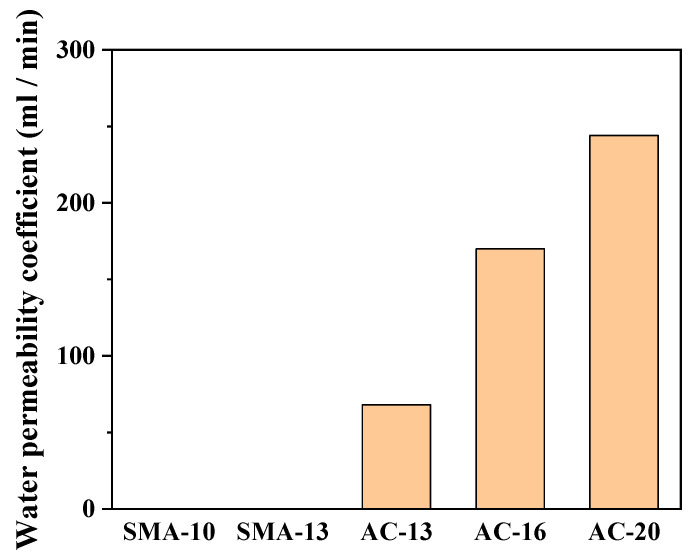
Water permeability coefficient results.

The permeability coefficient reflects the water-sealing capacity of asphalt mixtures and serves as a key indicator for evaluating the compactness of surface course structures. The test results are presented in Figure 9. Both SMA-10 and SMA-13 exhibited permeability coefficients of 0 mL/min, indicating a highly dense structure with excellent pore closure. This outstanding impermeability ensures the effective protection of underlying layers from moisture infiltration. AC-13 recorded a permeability coefficient of 68 mL/min, which, although significantly higher than that of SMA mixtures, still demonstrated notable advantages compared with coarser-graded mixtures. In contrast, AC-16 and AC-20 showed considerably higher permeability coefficients at 170 mL/min and 244 mL/min, respectively. This behavior is attributed to their coarser aggregate gradation and higher air void contents, which tend to form interconnected pore channels, thereby compromising water stability [18]. Accordingly, in regions characterized by frequent rainfall or high humidity, SMA is recommended as a surface course material to enhance pavement waterproofing performance and safeguard the durability of the overall structure.

### 3.3. Structural Optimization Design

#### 3.3.1. Rutting Test of Composite Specimens

**Figure 10:**
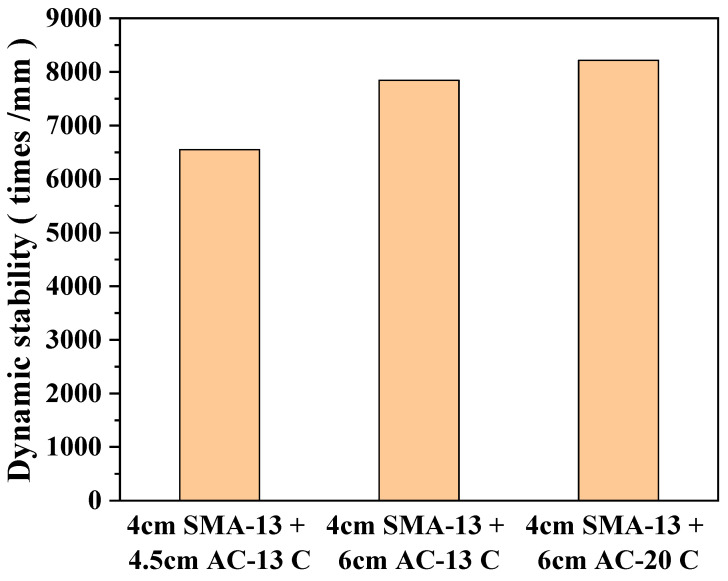
Rutting test results.

The high-temperature performance of composite rutting slabs with different structural combinations is summarized in Figure 10. Under identical gradation structures, specimen height had a significant effect on dynamic stability, specimens with a height of 10 cm exhibited markedly higher dynamic stability than the 8.5 cm specimens, with an average increase of approximately 1295 passes/mm. This demonstrates that increasing structural thickness contributes effectively to enhancing rutting resistance under high-temperature conditions. Furthermore, for slabs of the same thickness, the aggregate gradation of the lower layers significantly affects the overall structural performance. When coarse-graded AC-20C was used as the intermediate and lower layer material, the dynamic stability of the composite structure increased by 372 passes/mm compared with the combination using AC-13C, indicating that AC-20C is more effective at distributing high-temperature shear stresses and improving the structural stability of the system. However, for the “thin-layer structure” composed of a 4 cm surface course plus a 4.5 cm lower course, although this configuration offers certain advantages in terms of material saving and lightweight construction, its shear resistance under high temperatures is insufficient. This can lead to interlayer slippage and shear failure. Considering the extreme conditions in hot and humid regions, such as bridge deck temperatures frequently exceeding 60 °C in summer and exposure to repeated rainfall, further enhancement of the waterproofing and bonding performance of this thin-layer structure is recommended [19]. In particular, improvements in interfacial shear strength and high-temperature stability are critical to ensure the long-term reliability and durability of bridge deck pavements.

#### 3.3.2. Pull-Out Test

**Figure 11:**
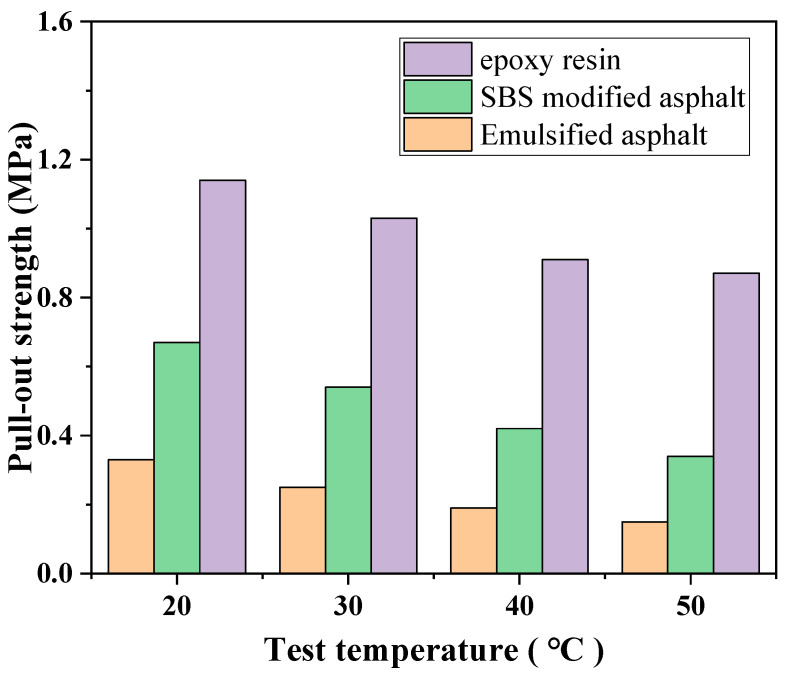
Pull-out test results.

To evaluate the interlayer bonding performance of different tack coat materials under varying temperature conditions, pull-off tests were conducted on emulsified asphalt, SBS-modified asphalt, and epoxy resin at 20 °C, 30 °C, 40 °C, and 50 °C. The test results at each temperature are presented in Figure 11. At room temperature (20 °C), the interlayer pull-off strength of the materials followed the order: epoxy resin (1.14 MPa) > SBS-modified asphalt (0.67 MPa) > emulsified asphalt (0.33 MPa). Epoxy resin exhibited significantly superior bonding performance compared to the other two materials, reflecting excellent interfacial adhesion and high structural stability. As temperature increased, the pull-off strength of all three materials showed a clear decreasing trend, indicating that elevated temperatures substantially weaken interfacial bonding. Notably, emulsified asphalt experienced the most pronounced decline, with pull-off strength dropping from 0.33 MPa at 20 °C to 0.15 MPa at 50 °C, a reduction of approximately 54.5%. This sensitivity is primarily due to its limited physical stability; heating significantly reduces its interfacial bonding capacity and leads to softening or damage within the binder layer. In contrast, SBS-modified asphalt showed a moderate decrease from 0.67 MPa to 0.34 MPa (about 49.3%), indicating lower but still notable temperature sensitivity. Epoxy resin demonstrated the strongest high-temperature performance, with strength decreasing only from 1.14 MPa to 0.87 MPa (approximately 23.7%), confirming its excellent thermal stability and reliable adhesion over a wide temperature range. Overall, epoxy resin tack coats exhibited the highest pull-off strength and the lowest temperature sensitivity, making them suitable for pavement structures in regions with large temperature variations or high-temperature conditions. Although emulsified asphalt offers a lower-cost alternative, its poor temperature stability necessitates cautious application in areas subject to significant thermal fluctuations.

#### 3.3.3. Pull-Out Test

**Figure 12:**
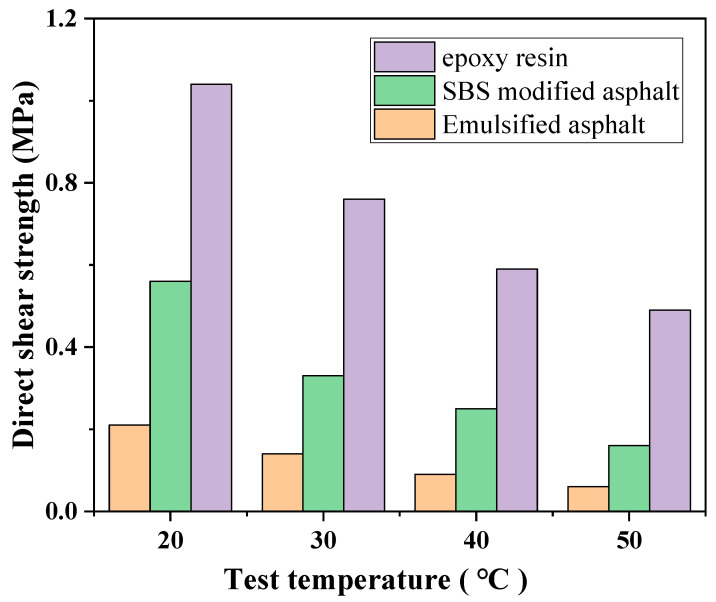
Direct shear test results.

To further evaluate the interlayer shear resistance, direct shear tests were conducted on the three coatings’ bond lines without normal pressure. The results are shown in Figure 12. Consistent with the pull-out test results, the direct shear strength of the three materials gradually decreased with increasing temperature within the temperature range of 20 °C to 50 °C, maintaining the strength ranking as follows: epoxy resin > SBS-modified asphalt > emulsified asphalt. This trend indicates that changes in direct shear strength are also controlled by interfacial bonding properties, rather than solely relying on frictional resistance caused by the rigidity or roughness of the materials themselves. The shear strength of emulsified asphalt decreased from 0.21 MPa at 20 °C to 0.06 MPa at 50 °C, a 71.4% decrease, the most dramatic of the three, highlighting its poor resistance to high-temperature shear failure. Meanwhile, the shear strength of SBS-modified asphalt decreased from 0.56 MPa to 0.16 MPa in the same temperature range, a decrease of approximately 71.4%, comparable to that of emulsified asphalt. This indicates that while this modified asphalt exhibits slightly superior pull-out strength, its shear resistance at high temperatures remains limited. In contrast, the shear strength of epoxy resin decreased from 1.04 MPa to 0.49 MPa, a drop of approximately 52.9%. Although affected by temperature, the drop was relatively mild, and its absolute values at all temperature points were significantly higher than those of the other two materials, further confirming its excellent interfacial bonding strength and shear resistance. It can be inferred that in areas significantly affected by high temperatures or in heavily loaded road structures, the use of epoxy resin coatings as bonding layers can effectively improve the shear and tensile properties between layers, enhancing the stability of the overall structure.

## 4. Conclusions

This study focused on concrete bridge deck pavement systems and conducted a systematic analysis and experimental investigation in three key areas: mixture proportioning design, pavement performance testing, and structural optimization. The objective was to develop a pavement structure with coordinated material performance, rational structural design, and suitability for service in hot and humid environments. The main conclusions are as follows:

(1) The experimental results indicate that SMA-13 exhibited excellent stability (11.39 kN) and an appropriate air void content (4.3%), making it suitable for use as a surface course on bridge decks. AC-13 provides a balance of compactness (air voids 4.8%) and structural stability (stability 11.04 kN), making it well-suited as an intermediate and lower layer. SMA-13 and AC-13 demonstrate good coordination in terms of density, binder saturation, and other key indices, providing a mechanically stable foundation for the upper and middle structural layers.
(2) Comprehensive testing verified the mixtures’ adaptability to service conditions. SMA-13 demonstrated outstanding high-temperature stability, with dynamic stability reaching 7132 passes/mm, significantly higher than AC-13 (5037 passes/mm). Its excellent water resistance was also evident, with a residual stability of 93.4% and a permeability coefficient of 0 mL/min, reflecting strong resistance to moisture damage and effective impermeability. At low temperatures, SMA-13 exhibited a bending strain of 3261 με, meeting the crack resistance requirements and confirming its overall superior pavement performance.
(3) Pull-off and direct shear tests showed that epoxy resin tack coats maintained high bonding strength across different temperatures (pull-off strength of 1.14 MPa at 20 °C, decreasing only to 0.87 MPa at 50 °C), outperforming SBS-modified asphalt and emulsified asphalt. This indicates strong thermal stability and reliable interlayer adhesion. Based on these results, a “SMA-13 + epoxy resin + AC-13” structural combination is recommended, forming a strong-yet-ductile, synergistic bridge deck pavement system.

In summary, the scientific mixture proportioning ensures a solid structural performance foundation, comprehensive pavement performance testing confirms service adaptability, and the rational structural optimization enhances overall safety and durability. The findings of this study provide reliable design guidance and practical reference for concrete bridge deck pavement engineering.

## Figures and Tables

**Table 1:** Properties of high-viscosity asphalt.

Properties	Unit	Test Results	Test Methods
Penetration (25 °C, 100 g, 5 s)	0.1 mm	48.4	ASTM D5
Softening point	°C	89.5	ASTM D36
Ductility (5 °C, 5 cm/min)	cm	33.7	ASTM D113

**Table 2:** Properties of aggregates.

Aggregate Size (mm)	9.5–13.2	4.75–9.5	2.36–4.75	0–2.36	Mineral Power
Apparent specific gravity (g/cm^3^)	2.913	2.934	2.927	2.876	2.746
Water absorption (%)	1.019	1.227	0.984	2.131	-
Los Angeles abrasion (%)	16.75	14.83	13.47	-	-
Crushing value (%)	12.52	-	-	-	-

**Table 3:** Aggregate gradation and mix compositions of asphalt mixtures.

Sieve Size (mm)	26.5	19	16	13.2	9.5	4.75	2.36	1.18	0.6	0.3	0.15	0.075
SMA-10	100	100	100	100	94.4	47.2	29.3	24.2	21.1	16.2	14.1	10.8
SMA-13	100	100	100	93.8	56.8	26.1	21.4	18	15.4	13.4	12.1	10.1
AC-13	100	100	100	98	59.2	33.4	25.1	17	11.6	8.7	7.2	6.2
AC-16	100	100	97	87.9	66.8	39.2	28.4	22.7	17.9	12.6	8.6	5.0
AC-20	100	98.1	85.9	71.5	60.3	36.5	26.3	19.4	14.3	9.0	6.7	5.2

**Table 4:** Marshall test results.

Grading Type	SMA-10	SMA-13	AC-13	AC-16	AC-20
Maximum theoretical relative density	2.653	2.559	2.507	2.479	2.548
Bulk density (g/cm^3^)	2.483	2.415	2.380	2.322	2.375
Void ratio (%)	3.5	4.3	4.8	6.9	7.2
Mineral void ratio (%)	16.5	16.5	15.7	16.7	13.6
Asphalt saturation (%)	75.17	74.60	66.40	56.31	46.30
Stability (kN)	9.22	11.39	11.04	8.55	9.35
Flow value (mm)	3.22	3.32	3.95	3.87	3.37

**Table 5:** Immersion Marshall test results.

Grading Type	SMA-10	SMA-13	AC-13	AC-16	AC-20
MS (KN)	9.22	11.39	11.04	8.55	9.35
MS1 (KN)	8.60	10.64	9.77	7.10	7.55
IRS (%)	93.2	93.4	88.5	83.0	80.7

## Data Availability

The raw data supporting the conclusions of this article will be made available by the authors on request.

## References

[1] Sha A., Jiang W., Shan J., Wu W., Li Y., Zhang S. (2022). Pavement structure and materials design for sea-crossing bridges and tunnel: Case study of the Hong Kong–Zhuhai–Macau Bridge. J. Road Eng..

[2] Abdal S., Mansour W., Agwa I., Nasr M., Abadel A., Onuralp Özkılıç Y., Akeed M.H. (2023). Application of ultra-high-performance concrete in bridge engineering: Current status, limitations, challenges, and future prospects. Buildings.

[3] Zhao R., Yuan Y., Wei X., Shen R., Zheng K., Qian Y., Pu Q., Zhang Q., Liao H., Li X. (2020). Review of annual progress of bridge engineering in 2019. Adv. Bridge Eng..

[4] Murata Y., Kariya K., Yashima A., Okamura T., Quan N.H., Yokota Y., Ito S., Tsuji S. (2020). Long-life repair method for road based on soundness evaluation of embankment and pavement. Jpn. Geotech. Soc. Spec. Publ..

[5] Angelini A. (2024). Life Cycle Assessment on Port Pavements: Case Study-Norvik Port. Master’ Thesis.

[6] Gaeta M.G., Martinelli L., Lamberti A., Lynett P.J., Smith J.M. Uplift forces on wave exposed jetties: Scale comparison and effect of venting. Proceedings of the 33rd International Conference on Coastal Engineering.

[7] Mouton Y. (2013). Organic Materials for Sustainable Civil Engineering.

[8] Shahnewaz S., Masri K.A., Ghani N. (2021). Porous asphalt modification using different types of additives: A review. Construction.

[9] Kelly R., Indraratna B., Powrie W., Zapata C., Kikuchi Y., Tutumluer E., Correia A. (2022). State of the Art on Transport Geotechnics. Proceedings of the 20th International Conference on Soil Mechanics and Geotechnical Engineering.

[10] Wang H.-J., Sun J.-Q., Chen H.-P., Zhu Y.-L., Zhang Y., Jiang D.-B., Lang X.-M., Fan K., Yu E.-T., Yang S. (2012). Extreme climate in China: Facts, simulation and projection. Meteorol. Z..

[11] Sun L. (2016). Structural Behavior of Asphalt Pavements: Intergrated Analysis and Design of Conventional and Heavy Duty Asphalt Pavement.

[12] Lu Q., Sha A., Jiang W., Jiao W., Chen Y., Feng Z., Wang S., Li Z. (2025). Cooling water-retentive semi-flexible pavement with light-colored grout and recycled materials. Constr. Build. Mater..

[13] Lu Q., Sha A., Chen Y., Liu Z., Feng Z., Li Z., Jiao W. (2025). Development of high absorbency and lightweight water-retentive semi-flexible pavement for urban heat island mitigation. Constr. Build. Mater..

[14] Wu H., Li P., Nian T., Zhang G., He T., Wei X. (2019). Evaluation of asphalt and asphalt mixtures’ water stability method under multiple freeze-thaw cycles. Constr. Build. Mater..

[15] Miranda H.M.B., Batista F.A., Neves J., de Lurdes Antunes M. (2021). Influence of the aggregate skeleton matrix and volumetric composition on the resistance of stone mastic asphalt to permanent deformation. Road Mater. Pavement Des..

[16] Zhang Z., Qian W., Wang S., Chen Y., Wang N., Zhao Q., Li H., Gao G., Zhao Y., Zhan H. (2024). Activating continuous dislocation pinning enhanced toughness of nanocomposite coating through specially oriented semicoherent heterointerface lattice distortion. Ceram. Int..

[17] Zhang M., Zhang J., Lyu L., Li Y., Tan X., Li Z., Pei J. (2024). Durable and environmental asphalt pavement with plant fiber: A state-of-the-art review. J. Mater. Civ. Eng..

[18] Song H., Fan S., Wan K., Yao J., Yin W., Lee Y. (2025). Balancing mechanical and permeability properties of pervious concrete through inter-aggregate pore structure optimization. J. Build. Eng..

[19] Lu Q., Sha A., Chen Y., Ren X., Wang S., Wu J., Jiao W. (2025). Mechanical and thermal performance of semi-flexible pavement incorporating foam concrete grout. Constr. Build. Mater..

